# Diisopropyl pyrazine-2,5-dicarboxyl­ate

**DOI:** 10.1107/S1600536810029314

**Published:** 2010-08-04

**Authors:** Xiao-Qing Zhang, Wen-Shi Wu, Xin-Yu Wang, Jian-Hua Ma

**Affiliations:** a College of Materials Science and Engineering, Huaqiao University, Xiamen, Fujian, 361021, People’s Republic of China

## Abstract

The mol­ecule of the title compound, C_12_H_16_N_2_O_4_, is located on an inversion center. The carboxyl­ate groups are twisted slightly with respect to the pyrazine ring, making a dihedral angle of 6.4 (3)°.

## Related literature

For related structures, see: Cockriel *et al.* (2008 ▶); Vishweshwar *et al.* (2004 ▶).
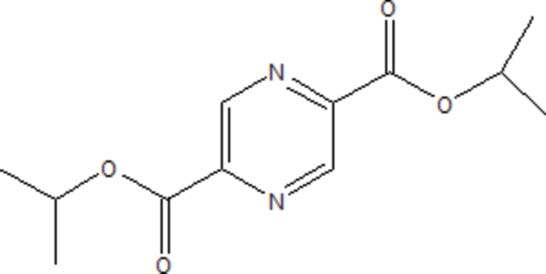


## Experimental

### 

#### Crystal data

C_12_H_16_N_2_O_4_
*M* *_r_* = 252.27
Monoclinic, 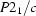

*a* = 4.7804 (1) Å
*b* = 15.6842 (3) Å
*c* = 9.1877 (2) Å
β = 104.227 (2)°
*V* = 667.74 (2) Å^3^
*Z* = 2
Mo *K*α radiation
μ = 0.10 mm^−1^
*T* = 296 K
0.44 × 0.20 × 0.09 mm

#### Data collection

Bruker P4 diffractometer
10015 measured reflections
1361 independent reflections
969 reflections with *I* > 2σ(*I*)
*R*_int_ = 0.028

#### Refinement

*R*[*F* ^2^ > 2σ(*F* ^2^)] = 0.048
*wR*(*F* ^2^) = 0.148
*S* = 1.07
1361 reflections
84 parameters
H-atom parameters constrained
Δρ_max_ = 0.22 e Å^−3^
Δρ_min_ = −0.16 e Å^−3^

### 

Data collection: *XSCANS* (Bruker, 1999 ▶); cell refinement: *XSCANS*; data reduction: *SHELXTL* (Sheldrick, 2008 ▶); program(s) used to solve structure: *SHELXS97* (Sheldrick, 2008 ▶); program(s) used to refine structure: *SHELXL97* (Sheldrick, 2008 ▶); molecular graphics: *SHELXTL*; software used to prepare material for publication: *SHELXTL*.

## Supplementary Material

Crystal structure: contains datablocks global, I. DOI: 10.1107/S1600536810029314/dn2583sup1.cif

Structure factors: contains datablocks I. DOI: 10.1107/S1600536810029314/dn2583Isup2.hkl

Additional supplementary materials:  crystallographic information; 3D view; checkCIF report

## Figures and Tables

**Fig. 1.**
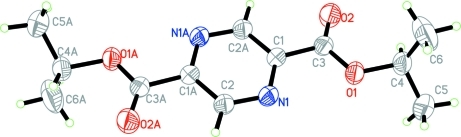
Molecular view of the title compound with the atom labeling scheme. Ellipsoids are drawn at the 30% probability level. [Symmetry code: (A) -x+1, -y+1, -z+1].

## supplementary crystallographic information

### Comment

The molecule of the title compound is is organized around inversion center (Fig. 1). The carboxylate group are slightly twisted with respect to the pyrazine ring making a dihedral angle of 6.4 (3)°.The carboxyl C—O and C═ O bonds are normal, while the bond angle of C—N═C are slightly smaller than those in pyrazine-2,5-dicarboxylic acid dihydrate (Vishweshwar *et al.*,2004). The angle C3—O1—C4 of 117.60 (14) is larger compared to the value of 115.04 (16) in Pyrazine-2,5-dicarboxylic acid dimethyl ester (Cockriel *et al.*, 2008). The atoms of O(1) to C(5) may be considered to control the molecular packing through intermolecular hydrophobic interaction of the isopropyl groups. The crystal structure is stabilized *via* van der Waals forces.

### Experimental

The title compound was synthesized by dissolving 2,5-pyrazinedicarboxylic acid (200 mg,11.9 mmol)in 200 ml 2-propanol, while stirring 2 ml concentrated H_2_SO_4_ was added slowly.The solution was left to reflux for 12 h, then distillation under reduced pressure until no solution to outflow after filtered.The solution was made neutral with Na_2_CO_3_(aq), extracted with 30 ml e thyl acetate.Orange crystals of the title compound would be grew by slow evaporating at room temperature after five days.

### Refinement

The C-bound H atoms were included in the riding model approximation with C—H=0.93, all these H atoms included in the final refinement. The *U*_iso_ of each H atom = 1.2*U*_eq_(C). The *U*_eq_ of C4 is regular. The checkcif considers the *U*_eq_ of C4 is low, this is because it is lower compared with the C5 and C6.

### Crystal data

**Table:**

C_12_H_16_N_2_O_4_	*F*(000) = 268
*M**_r_* = 252.27	*D*_x_ = 1.255 Mg m^−^^3^
Monoclinic, *P*2_1_/*c*	Mo *K*α radiation, λ = 0.71073 Å
Hall symbol: -P 2ybc	Cell parameters from 1552 reflections
*a* = 4.7804 (1) Å	θ = 2.6–27.7°
*b* = 15.6842 (3) Å	µ = 0.10 mm^−^^1^
*c* = 9.1877 (2) Å	*T* = 296 K
β = 104.227 (2)°	Block, orange
*V* = 667.74 (2) Å^3^	0.44 × 0.20 × 0.09 mm
*Z* = 2	

### Data collection

**Table:**

Bruker P4 diffractometer	969 reflections with *I* > 2σ(*I*)
Radiation source: fine-focus sealed tube	*R*_int_ = 0.028
graphite	θ_max_ = 26.4°, θ_min_ = 2.6°
Detector resolution: 0 pixels mm^-1^	*h* = −5→5
ω scans	*k* = 0→19
10015 measured reflections	*l* = 0→11
1361 independent reflections	

### Refinement

**Table:**

Refinement on *F*^2^	Primary atom site location: structure-invariant direct methods
Least-squares matrix: full	Secondary atom site location: difference Fourier map
*R*[*F*^2^ > 2σ(*F*^2^)] = 0.048	Hydrogen site location: inferred from neighbouring sites
*wR*(*F*^2^) = 0.148	H-atom parameters constrained
*S* = 1.07	*w* = 1/[σ^2^(*F*_o_^2^) + (0.0658*P*)^2^ + 0.1415*P*] where *P* = (*F*_o_^2^ + 2*F*_c_^2^)/3
1361 reflections	(Δ/σ)_max_ < 0.001
84 parameters	Δρ_max_ = 0.22 e Å^−^^3^
0 restraints	Δρ_min_ = −0.16 e Å^−^^3^

### Special details

**Table:**

Geometry. All e.s.d.'s (except the e.s.d. in the dihedral angle between two l.s. planes) are estimated using the full covariance matrix. The cell e.s.d.'s are taken into account individually in the estimation of e.s.d.'s in distances, angles and torsion angles; correlations between e.s.d.'s in cell parameters are only used when they are defined by crystal symmetry. An approximate (isotropic) treatment of cell e.s.d.'s is used for estimating e.s.d.'s involving l.s. planes.
Refinement. Refinement of *F*^2^ against ALL reflections. The weighted *R*-factor *wR* and goodness of fit *S* are based on *F*^2^, conventional *R*-factors *R* are based on *F*, with *F* set to zero for negative *F*^2^. The threshold expression of *F*^2^ > σ(*F*^2^) is used only for calculating *R*-factors(gt) *etc*. and is not relevant to the choice of reflections for refinement. *R*-factors based on *F*^2^ are statistically about twice as large as those based on *F*, and *R*- factors based on ALL data will be even larger.

### Fractional atomic coordinates and isotropic or equivalent isotropic displacement parameters (Å^2^)

**Table:**

	*x*	*y*	*z*	*U*_iso_*/*U*_eq_	
O1	0.2207 (3)	0.61807 (8)	0.76556 (16)	0.0760 (5)	
O2	0.0875 (4)	0.48234 (10)	0.7742 (2)	0.0932 (6)	
N1	0.4741 (4)	0.58450 (9)	0.54265 (18)	0.0671 (5)	
C1	0.3667 (4)	0.52028 (10)	0.6052 (2)	0.0563 (5)	
C2	0.6071 (4)	0.56289 (12)	0.4371 (2)	0.0683 (5)	
H2A	0.6858	0.6058	0.3898	0.082*	
C3	0.2104 (4)	0.53794 (12)	0.7251 (2)	0.0625 (5)	
C4	0.0807 (5)	0.64177 (14)	0.8860 (2)	0.0803 (6)	
H4A	−0.0748	0.6011	0.8864	0.096*	
C5	−0.0461 (8)	0.72695 (18)	0.8468 (4)	0.1229 (11)	
H5A	−0.1475	0.7443	0.9200	0.184*	
H5B	−0.1781	0.7249	0.7494	0.184*	
H5C	0.1046	0.7672	0.8454	0.184*	
C6	0.2971 (8)	0.6359 (3)	1.0299 (3)	0.1457 (15)	
H6A	0.2050	0.6437	1.1108	0.219*	
H6B	0.4403	0.6795	1.0346	0.219*	
H6C	0.3877	0.5809	1.0384	0.219*	

### Atomic displacement parameters (Å^2^)

**Table:**

	*U*^11^	*U*^22^	*U*^33^	*U*^12^	*U*^13^	*U*^23^
O1	0.0981 (11)	0.0603 (8)	0.0829 (9)	−0.0050 (7)	0.0476 (8)	−0.0104 (6)
O2	0.1167 (13)	0.0701 (9)	0.1115 (13)	−0.0127 (8)	0.0640 (11)	−0.0055 (8)
N1	0.0815 (11)	0.0508 (8)	0.0747 (10)	−0.0003 (7)	0.0303 (8)	−0.0029 (7)
C1	0.0563 (10)	0.0523 (9)	0.0600 (10)	0.0013 (7)	0.0139 (8)	−0.0013 (7)
C2	0.0830 (13)	0.0535 (10)	0.0758 (12)	−0.0043 (9)	0.0334 (11)	−0.0006 (9)
C3	0.0650 (11)	0.0580 (10)	0.0671 (11)	0.0028 (8)	0.0209 (9)	0.0002 (8)
C4	0.1000 (16)	0.0706 (12)	0.0855 (15)	−0.0042 (11)	0.0519 (13)	−0.0096 (10)
C5	0.173 (3)	0.0914 (18)	0.124 (2)	0.0344 (19)	0.075 (2)	−0.0047 (16)
C6	0.141 (3)	0.231 (4)	0.0736 (17)	0.040 (3)	0.0424 (18)	0.000 (2)

### Geometric parameters (Å, °)

**Table:**

O1—C3	1.308 (2)	C4—C5	1.475 (4)
O1—C4	1.474 (2)	C4—H4A	0.9800
O2—C3	1.200 (2)	C5—H5A	0.9600
N1—C1	1.324 (2)	C5—H5B	0.9600
N1—C2	1.327 (2)	C5—H5C	0.9600
C1—C2^i^	1.376 (2)	C6—H6A	0.9600
C1—C3	1.500 (3)	C6—H6B	0.9600
C2—H2A	0.9300	C6—H6C	0.9600
C4—C6	1.468 (4)		
			
C3—O1—C4	117.61 (15)	O1—C4—H4A	108.9
C1—N1—C2	115.43 (15)	C5—C4—H4A	108.9
N1—C1—C2^i^	121.76 (17)	C4—C5—H5A	109.5
N1—C1—C3	119.62 (15)	C4—C5—H5B	109.5
C2^i^—C1—C3	118.62 (16)	H5A—C5—H5B	109.5
N1—C2—C1^i^	122.82 (17)	C4—C5—H5C	109.5
N1—C2—H2A	118.6	H5A—C5—H5C	109.5
C1^i^—C2—H2A	118.6	H5B—C5—H5C	109.5
O2—C3—O1	125.35 (18)	C4—C6—H6A	109.5
O2—C3—C1	121.35 (17)	C4—C6—H6B	109.5
O1—C3—C1	113.29 (16)	H6A—C6—H6B	109.5
C6—C4—O1	108.1 (2)	C4—C6—H6C	109.5
C6—C4—C5	115.6 (3)	H6A—C6—H6C	109.5
O1—C4—C5	106.28 (18)	H6B—C6—H6C	109.5
C6—C4—H4A	108.9		
			
N1—C1—C3—O1	−6.0 (3)		

Symmetry codes: (i) −*x*+1, −*y*+1, −*z*+1.
